# Dietary linoleic acid and the ratio of unsaturated to saturated fatty acids are inversely associated with significant liver fibrosis risk: A nationwide survey

**DOI:** 10.3389/fnut.2022.938645

**Published:** 2022-07-26

**Authors:** Tingting Zhu, Xiao-Ting Lu, Zhao-Yan Liu, Hui-Lian Zhu

**Affiliations:** ^1^Department of Nutrition, School of Public Health, Sun Yat-sen University, Guangzhou, China; ^2^Guangdong Provincial Key Laboratory of Food, Nutrition and Health, School of Public Health, Sun Yat-sen University, Guangzhou, China; ^3^Department of Food Science and Engineering, School of Food Science and Engineering, Hainan Tropical Ocean University, Sanya, China; ^4^Collaborative Innovation Center of Provincial and Ministerial Co-construction for Marine Food Deep Processing, Hainan Tropical Ocean University, Sanya, China

**Keywords:** dietary fatty acids, ratio of unsaturated to saturated fatty acids, dietary fatty acid components, significant liver fibrosis, nationwide study

## Abstract

Since no pharmaceuticals have been proven to effectively reduce liver fibrosis, dietary fatty acids may be beneficial as one of the non-pharmaceutical interventions due to their important roles in liver metabolism. In this cross-sectional study, we analyzed the data from the 2017–2018 cycle of National Health and Nutrition Examination Survey to examine the associations between the proportion and composition of dietary fatty acid intakes with significant liver fibrosis among US population. The dietary fatty acid consumptions were calculated based on two 24-h dietary recalls. Significant liver fibrosis was diagnosed based on liver stiffness measurement value derived from the vibration controlled transient elastography. Multivariate logistic regression analysis and sensitivity analysis were performed to assess the association between dietary fatty acid consumption and significant liver fibrosis risk. Finally, restricted cubic spline analysis was carried out to explore the dose–response between polyunsaturated fatty acids (PUFA) or linoleic acid intakes and the risk of significant liver fibrosis. The results showed that the multivariate adjusted odds ratios (95% confidence intervals) of significant liver fibrosis were 0.34 (0.14–0.84), 0.68 (0.50–0.91), and 0.64 (0.47–0.87) for the highest level of unsaturated to saturated fatty acid ratio, dietary PUFA, and linoleic acid intakes compared to the lowest reference, respectively. The sensitivity analysis and restricted cubic spline analysis produced similar results, reinforcing the inverse association of unsaturated to saturated fatty acid ratio, PUFA, and linoleic acid consumptions with significant liver fibrosis risk. However, other dietary fatty acids did not show the statistically significant association with significant liver fibrosis. In conclusion, dietary linoleic acid may play a key role in the inverse association between the unsaturated to saturated fatty acid ratio and the risk of significant liver fibrosis. Further studies are needed to confirm these findings.

## Introduction

Liver fibrosis, the result of wound healing response to chronic liver injury (1), is prevalent worldwide and can be related to the kinds of chronic liver diseases (CLD) (2). Furthermore, liver fibrosis is known as the main reason for liver disease-related morbidity and mortality (3). Among the CLD, non-alcoholic fatty liver disease (NAFLD) is a representative one, which contains a series of proceeding liver damages, ranging from simple hepatic steatosis to non-alcoholic steatohepatitis and fibrosis, cirrhosis, and even cancer (4). NAFLD-related advanced fibrosis has been reported to have an accelerated increasing trend in the US population (5). Large-scale observational studies have demonstrated that a progressive stage of fibrosis, ranging from significant fibrosis to cirrhosis, is the most powerful histological predictor of hepatic all-cause mortality in NAFLD (6, 7). In addition, the development of liver fibrosis into a more progressive stage mainly occurs when existing chronically liver damage due to infectious, metabolic, toxic/drug-induced, cholestatic, or autoimmune insult (8). Since there are still no approved antifibrotic pharmaceuticals for liver fibrosis (8), the non-medical elements are critical to delaying or even reversing the progression of liver fibrosis. Cost-effective modifiable dietary nutrients are considered to be one of them.

The liver is an important organ for the metabolic regulation of dietary fat, 15% of liver triacylglycerol comes from the diet (9). Among the dietary fat, the fatty acid compositions are relevant to hepatic lipogenesis because of their different metabolic and functional activities (10). The dietary fatty acid compositions can be distinguished by both degrees of the number of carbon atoms and configuration of the saturation (11). Based on the number of double bonds, saturated fatty acids (SFA), monounsaturated fatty acids (MUFA), and polyunsaturated fatty acids (PUFA), which are further sub-categorized into their specific fatty acid components.

Dietary fatty acids can regulate the distribution of fat in the human body, independent of body weight change (12), and take part in the metabolic pathways (13, 14). According to the results of a randomized controlled trial, a hypercaloric SFA-rich diet led to a remarkable increase in hepatic fat; by contrast, a PUFA-enriched diet did not increase hepatic fat, albeit similar weight gain in both groups (12). Several studies have also observed that SFA can induce endoplasmic reticulum stress and result in liver damage (15, 16), whereas n-3 PUFA showed protective activities to the pathological conditions in NAFLD, macrosteatotic livers, and acute hepatitis (17–19). Nevertheless, contradictory results were also found. The subjects with human immunodeficiency virus (HIV) were reported to be lower odds of having liver fibrosis when consuming lauric and myristic SFA intermediately (20). Additionally, the mice fed with additional eicosapentaenoic (EPA) and docosahexaenoic (DHA) acids represented high expression of tissue inhibitor of metalloproteinase (TIMP)-1 and transforming growth factor (TGF)-β profibrogenic genes, and more severe fibrosis score (21).

Based on the contradictory results of PUFA, SFA, and their specific components, it is thus necessary to advance our understanding of the association of dietary fatty acids with liver fibrosis, especially for the specific fatty acid components. However, to our knowledge, there are sparse epidemiologic studies assessing the associations between specific fatty acid components and liver fibrosis in a large-scale population that is representative nationally, possibly due to the lack of suitable screening techniques for liver fibrosis among such large-scale population (22). Liver biopsy, as the gold standard for liver fibrosis evaluation, with the shortcoming of invasiveness, poor acceptability, not-easy handling, and so on, is not well-suitable for the large-scale population survey. Until the appearance of vibration controlled transient elastography (VCTE), with the advantages of non-invasiveness, better acceptability, and accurate technique, VCTE has been widely used as the non-invasive standard tool for evaluating hepatic fibrosis by liver stiffness measurement (LSM) (23). VCTE was first used as a part of the survey process in the 2017–2018 cycle of National Health and Nutrition Examination Survey (NHANES) (24). Using the more accessible and accurate diagnostic technique will provide a valid assessment of the population-based burden of liver fibrosis in the United States. Moreover, 2015–2020 dietary guidelines for Americans recommend that adults keep within saturated fat limits and replace SFA with unsaturated fatty acids. The ratio of unsaturated fatty acids (UFA) to SFA was first added to healthy eating index-2010 (HEI-2010) to evaluate diet and retained in the HEI-2015. However, scarce epidemiologic studies have referred to the ratio of UFA to SFA. Therefore, herein, we tried to estimate whether the components of dietary fatty acids or the ratio of UFA to SFA were associated with significant liver fibrosis assessed by VCTE among US adults.

## Materials and methods

### Data source

The cross-sectional study was conducted using the data from the 2017–2018 cycle of NHANES, which can be attained on the NHANES website (http://www.cdc.gov/nchs/nhanes.htm). The NHANES data are a multi-stage, stratified, cluster sample representative of the US non-institutionalized civilians (24). The data collection and methodology of NHANES have been reported in detail previously (25). Briefly, NHANES is comprised of questionnaires to obtain the demographic, socioeconomic, dietary, health-associated information, and a standardized physical examination to obtain the equipment-needed indexes. The National Center for Health Statistics Research Ethics Review Board approved the protocol of NHANES and all participants have provided written informed consent before data collection.

### Study population and design

**Figure 1:**
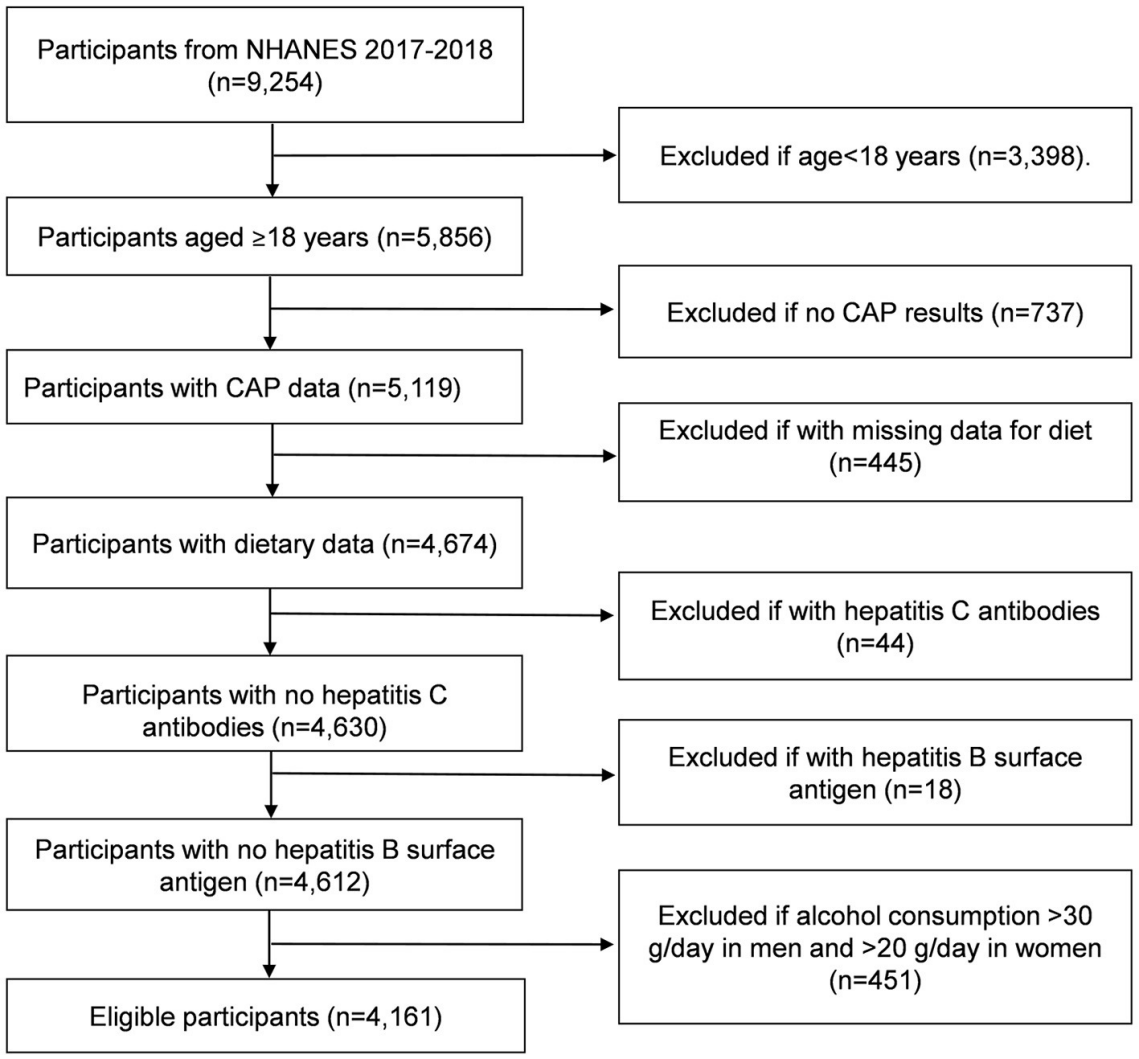
Flowchart of participants from 2017 to 2018 cycle of National Health and Nutrition Examination Survey (NHANES). CAP, controlled attenuation parameter.

The participants with age older than 18 years in the 2017–2018 NHANES cycle (n=5,856) and finished both the survey and medical examination were included. We excluded the participants if they did not have complete VCTE data (*n* = 737) and dietary data (*n* = 445). We also excluded the participants if they were examined with the presence of hepatitis C antibodies (*n* = 44) and hepatitis B surface antigen (*n* = 18), and if they had significant consumptions of alcohol (>30 g/d in men and >20 g/d in women) (*n* = 451). The final enrolled participants were 4,161 (Figure 1).

### Definition of significant liver fibrosis

Liver fibrosis was assessed using LSM data derived from VCTE with controlled attenuation, which was performed in the NHANES Mobile Examination Center (MEC). The VCTE measurements were taken using FibroScan^®^ model 502 V2 Touch (Echosens, Paris, France). The equipment can simultaneously measure the ultrasound attenuation and record the controlled attenuation parameter (CAP). CAP can be calculated only if the LSM is valid. The detailed VCTE examination procedure has been reported previously (26).

The liver stiffness is derived by wave velocity when it passes through the liver tissue with 50 Hz by mechanical vibration. Complete examination should meet the conditions: fasting time of at least 3 h, 10, or more complete LSM (E), and liver stiffness interquartile range/ median E <30% (27). LSM ranges from 1.5 to 75 kPa, with higher values indicating more severe fibrosis (24). According to the previous studies (28, 29), LSM value higher than 8 kPa derived from VCTE was considered as significant liver fibrosis.

### Diet intake assessment

In the NHANES study, the daily average intakes of total energy, total fat, and dietary fatty acids were calculated based on two 24-h dietary recalls. The first dietary recall was administered in person at the NHANES MEC, and the second dietary recall was administered over the telephone 3 to 10 days later, which were conducted by trained interviewers. If the subjects did not complete the second dietary recall interview, only the first dietary recall was used as the average value. Quality control was used for completeness of recalls, missing information, inconsistent reports, and unclear notes. The ratio of UFA to SFA, which was first listed in HEI-2010 and retained in HEI-2015, was calculated as (PUFA + MUFA)/SFA. In terms of n-3 and n-6 PUFA, due to no specific classification of linolenic acid in NHANES (30), it mainly includes alpha-linolenic acid (n-3 PUFA) and a small part of gamma-linolenic acid (n-6 PUFA). Therefore, in this study, linolenic acid, together with stearidonic acid, eicosapentaenoic acid, clupanodonic acid, and docosahexaenoic acid were defined as n-3 PUFA, and linoleic acid and arachidonic acid were defined as n-6 PUFA. Additionally, aligned with the 2015–2020 Dietary Guidelines for Americans (DGAs), the HEI-2015 includes 13 components that sum to score of 100 in maximum to evaluate dietary quality (31).

### Collection of covariates

The following variates were evaluated for each participant: [1] Demographic information including age, gender (male, female), family income-to-poverty ratio (<1.0, 1.0–3.0, >3.0), education levels (less than high school, high school or equivalent, college or above), marital status (married/living with partner, widowed/divorced/separated, never married), ethnicity (non-Hispanic white, non-Hispanic black, Mexican American, and others) were collected. [2] Laboratory parameters including platelet count, alanine aminotransferase (ALT), aspartate aminotransferase (AST), gamma-glutamyl-transferase (GGT), alkaline phosphatase (ALP), albumin, and total bilirubin were tested. The methods to assessing laboratory parameters have been described elsewhere in detail (32). [3] Medical conditions. Prevalent pre-hypertension was defined systolic blood pressure between 120 and 139 mmHg or diastolic blood pressure between 80 and 89 mmHg. Prevalent hypertension was diagnosed as systolic blood pressure ≥140 mmHg or diastolic blood pressure ≥90 mmHg or taking antihypertensive medications by self-report. Prevalent prediabetes was defined as without diabetes mellitus, but with fasting plasma glucose level of 100 to 125 mg/dl, or 2-h plasma glucose level of 140 to 199 mg/dl, or glycated hemoglobin A1c (HbA1c) level of 5.7 to 6.4% or prediabetes diagnosis by self-report. Diabetes mellitus was defined as a fasting plasma glucose level ≥126 mg/dl, HbA1c level ≥6.5%, and/or use of a hypoglycemic agent or insulin or self-reported diabetes diagnosis. Prevalent cardiovascular disease (CVD) was defined if with the condition of coronary heart disease, stroke, angina, heart attack, or congestive heart failure by self-report. History of cancer was defined as self-reported physician diagnosis of any kind of cancer during the lifetime. Prevalent dyslipidemia was defined if total cholesterol ≥200 mg/dl, or triglyceride ≥150 mg/dl, or low-density lipoprotein-cholesterol ≥130 mg/dl, or high-density lipoprotein-cholesterol <40 mg/dl for men, high-density lipoprotein-cholesterol <50 mg/dl for women, or self-reported use of prescribed lipid-modifying medication. Those who took oral corticosteroid over 180 days were defined as having used oral corticosteroid. Depression was evaluated by the Patient Health Questionnaire (PHQ-9). We categorized depression status as less depression (0–4), mild depression (5–9), and major depression (≥10) according to the PHQ-9 score (33). [4] Body measurement and lifestyle factors. We defined current smokers as the participants who reported having smoked at least 100 cigarettes in their lifetime and still kept the habit of smoking at the time of the interview. Former smokers were those who had quit smoking before the interview. Non-smokers were those who smoked <100 cigarettes during their lifetime. Height, weight, and waist circumference were measured, and body mass index (BMI) was defined as measured weight in kilograms divided by measured height in meters squared. The sleep status was evaluated by sleep duration at night and self-reported sleep disorder. Regular exercise was defined as continuous exercise in moderate or vigorous intensity for at least 10 min in a typical week, causing an increase in breathing or heart rate at varying degrees.

### Statistical analysis

Because of a complex, multi-stage, cluster-sampling design applied by NHANES, we conducted appropriate sample weights to constitute representative population-level data for the US civilian (34). Demographic information, laboratory parameters, medical conditions and body measurement, lifestyle factors, and dietary information were presented as mean ± standard error (SE) for continuous variables and counts (weighted frequencies) for categorical variables in the baseline.

The dietary fatty acid intakes including UFA, SFA, PUFA, MUFA, n-3, n-6 PUFA, and their specific components were analyzed by energy-adjusted method (35). We used multivariate logistic regression model to consider the association between dietary fatty acid intakes and significant liver fibrosis risk. Model 1 was only adjusted for age and gender. Model 2 was adjusted for age, sex, family income-to-poverty ratio, education level, marital status, ethnicity, and laboratory parameters including platelet count, ALT, AST, GGT, ALP, albumin, total bilirubin, medical conditions including pre-hypertension, hypertension, diabetes, prediabetes, CVD, history of cancer, dyslipidemia, use of oral corticosteroid over 180 days, depression status, body measurement and life style factors including smoking status, BMI, waist circumference, regular exercise, HEI-2015, energy intake, sleep duration, and history of sleep disorders. The results were presented with odds ratios (ORs) and corresponding 95% confidence intervals (CIs).

With the consideration of the higher risk of liver fibrosis in those with history of cancer and with use of oral corticosteroid over 180 days, sensitivity analyses were performed by excluding the participants with history of cancer without or with oral corticosteroid administration over 180 days, respectively. Furthermore, after confirming the non-linear relationship of significant liver fibrosis with PUFA and linoleic acid, we used restricted cubic spline (RCS) with 4 knots located at the 5, 35, 65, and 95th centiles to flexibly model the association of PUFA or linoleic acid with significant liver fibrosis risk, with the same adjusted variables as those in the multiple logistic regression model 2. Data were analyzed using the R software 4.1.2 (R Foundation Vienna, Austria), SPSS version 20.0 for Windows (SPSS Inc., Chicago, IL, USA) and the GraphPad Prism 7.0 (La Jolla, California), considering *p*-value <0.05 to be statistically significant.

## Results

### Study characteristics

Among 9,254 participants from NHANES 2017–2018 cycle, 4,161 participants were enrolled. The flowchart of study population is shown in Figure 1. Table 1 describes the baseline characteristics of the enrolled participants, including demographic information, laboratory parameters, medical conditions and body measurement, life style factors, and dietary information. In brief, the participants included 48.6% male, with average age of 47.5 ± 0.8 years. Other demographic variates showed that 10.4% participants were without high school education and 18.1% participants were without marriage. Of note, participants had the higher BMI of 29.8 ± 0.3 kg/m^2^ and the higher waist circumference of 100.7 ± 0.8 cm. Additionally, 65.0% participants endured dyslipidemia. As for dietary intakes, energy intake was 2,040.4 ± 16.5 kcal/day, and the mean dietary SFA, UFA, MUFA, PUFA, n-3 PUFA, and n-6 PUFA are also listed in Table 1, respectively. With regard to the ratio of UFA to SFA, most participants ranged from 1.2 to 2.5 (76.1%).

**Table 1:** Baseline characteristics of the participants.

**Variables**	**All (*****n*** = **4,161)**
**Demographic information**	
Age, years	47.5 ± 0.8
Sex (Male), *n* (%)	2,015 (48.6)
**Family income-to-poverty ratio, *n* (%)**	
<1.0	642 (12.1)
1.0–3.0	1,727 (38.6)
>3.0	1,290 (49.3)
**Education levels, *n* (%)**	
Less than high school	753 (10.4)
High school or equivalent	956 (27.0)
College or above	2,225 (58.7)
**Marital status, *n* (%)**	
Married/living with partner	2,340 (60.0)
Widowed/divorced/separated	885 (18.0)
Never married	711 (18.1)
**Ethnicity, *n* (%)**	
Non-Hispanic white	1,442 (62.6)
Non-Hispanic black	971 (11.4)
Mexican American	582 (9.1)
Others	1,166 (17.0)
**Laboratory parameters**	
Platelet count, 10^9^/L	245.4 ± 2.4
ALT, IU/L	22.5 ± 0.3
AST, IU/L	21.5 ± 0.2
GGT, IU/L	27.8 ± 0.6
ALP, IU/L	77.4 ± 0.7
Albumin, g/L	41.0 ± 0.2
Total Bilirubin, μmol/L	8.1 ± 0.1
**Medical conditions**	
Prevalent pre-hypertension, *n* (%)	905 (23.1)
Prevalent hypertension, *n* (%)	1,854 (38.3)
Prevalent prediabetes, *n* (%)	1,588 (39.0)
Prevalent diabetes, *n* (%)	877 (15.4)
Prevalent CVD, *n* (%)	442 (8.3)
History of cancer, *n* (%)	415 (10.6)
Dyslipidemia, *n* (%)	2,753 (65.0)
Use of oral corticosteroid ≥180 days, *n* (%)	43 (0.9)
**PHQ-9 score, *n* (%)**	
0–4	3,163 (76.9)
5–9	649 (15.0)
≥10	349 (8.1)
**Body measurement and life style factors**	
BMI, kg/m^2^	29.8 ± 0.3
Waist circumference, cm	100.7 ± 0.8
**Smoking status, *n* (%)**	
Non-smoker	2,517 (60.2)
Former smoker	958 (24.1)
Current smoker	686 (15.7)
Regular exercise, *n* (%)	1,980 (53.8)
Sleep duration <8 h/day, *n* (%)	2,143 (54.3)
History of sleep disorder, *n* (%)	1,140 (29.2)
**Dietary information**	
HEI-2015	51.2 ± 0.8
Energy intake, kcal/day	2040.4 ± 16.5
**Ratio of UFA to SFA, *n* (%)**	
≤ 1.2	453 (12.0)
1.2–2.5	3,070 (76.1)
≥2.5	637 (12.0)
Total fat, g/day	73.95 ± 0.45
SFA, g/day	24.12 ± 0.27
UFA, g/day	42.55 ± 0.32
MUFA, g/day	25.34 ± 0.20
PUFA, g/day	17.20 ± 0.19
n-3 PUFA, g/day	1.73 ± 0.03
n-6 PUFA, g/day	15.38 ± 0.16

*Data were expressed as mean ± SE for continuous variables or as n (weighted %) for categorical variables*.

*ALT, alanine aminotransferase; AST, aspartate aminotransferase; GGT, gamma-glutamyl transferase; ALP, alkaline phosphatase; CVD, cardiovascular disease; PHQ-9, Patient Health Questionnaire; BMI, body mass index; HEI-2015, healthy eating index-2015; UFA, unsaturated fatty acid; SFA, saturated fatty acid; MUFA, monounsaturated fatty acid; PUFA, polyunsaturated fatty acid*.

### Multivariate analysis and sensitivity analysis

We used the multivariate logistic regression model to explore the associations between dietary fatty acid intakes and the risk of significant liver fibrosis. Higher UFA to SFA ratio was inversely associated with significant liver fibrosis risk. Specifically, the ORs (95% CIs) of significant liver fibrosis were 0.47 (0.25–0.88) in model 1 and 0.34 (0.14–0.84) in model 2 for the ratio of UFA to SFA (≥2.5) vs. the reference; 0.54 (0.34–0.87) in model 1 and 0.47 (0.29–0.74) in model 2 for the ratio of UFA to SFA (1.2–2.5) when compared to the reference, respectively (Table 2). The ORs (95% CIs) of significant liver fibrosis based on tertiles of SFA, UFA, MUFA, PUFA, n-3, and n-6 PUFA are also presented in Table 2. The ORs (95% CIs) of significant liver fibrosis for the highest tertile vs. the reference tertile were 0.74 (0.57–0.95) in model 1 and 0.68 (0.50–0.91) in model 2 for PUFA intake and 0.70 (0.53–0.93) in model 1 and 0.64 (0.47–0.88) in model 2 for n-6 PUFA intake. Additionally, the ORs (95% CIs) of significant liver fibrosis were 1.49 (1.03–2.18) for the highest tertile of SFA intake vs. lowest tertile in only age and gender adjusted model 1, which

**Table 2:** Multivariate logistic regression model considering dietary fatty acid intakes and the risk of significant liver fibrosis in participants, NHANES 2017–2018 (*n* = 4161).

	**Significant liver fibrosis**			
	**OR1** **(95% CI)** ^a^	***p*** **-value**	**OR2** **(95% CI)** ^b^	***p*** **-value**
**Ratio of UFA to SFA**				
≤ 1.2	1.00		1.00	
1.2–2.5	0.54 (0.34–0.87)	0.014	0.47 (0.29–0.74)	0.003
≥2.5	0.47 (0.25–0.88)	0.021	0.34 (0.14–0.84)	0.023
**Total fat, g/day**				
T1 ( ≤ 66.23)	1.00		1.00	
T2 (66.24–78.94)	0.93 (0.62–1.39)	0.706	1.08 (0.67–1.75)	0.723
T3 (≥78.95)	1.21 (0.86–1.71)	0.248	1.08 (0.71–1.66)	0.698
**SFA, g/day**				
T1 ( ≤ 19.99)	1.00		1.00	
T2 (20.00–25.58)	0.92 (0.57–1.48)	0.707	0.90 (0.40–2.00)	0.780
T3 (≥25.59)	1.49 (1.03–2.18)	0.038	1.47 (0.83–2.63)	0.175
UFA, g/day				
T1 ( ≤ 37.13)	1.00		1.00	
T2 (37.14–46.06)	0.91 (0.64–1.30)	0.569	0.91 (0.59–1.40)	0.636
T3 (≥46.07)	1.06 (0.77–1.44)	0.718	0.98 (0.66–1.45)	0.900
**MUFA, g/day**				
T1 ( ≤ 22.06)	1.00		1.00	
T2 (22.07–27.28)	1.09 (0.68–1.75)	0.701	1.14 (0.64–2.01)	0.643
T3 (≥27.29)	1.32 (0.90–1.93)	0.138	1.18 (0.77–1.81)	0.433
**PUFA, g/day**				
T1 ( ≤ 14.27)	1.00		1.00	
T2 (14.28–19.14)	1.10 (0.78–1.55)	0.561	1.13 (0.74–1.74)	0.550
T3 (≥19.15)	0.74 (0.57–0.95)	0.021	0.68 (0.50–0.91)	0.012
**n−3 PUFA, g/day**				
T1 ( ≤ 1.34)	1.00		1.00	
T2 (1.35–1.91)	1.02 (0.75–1.39)	0.871	1.04 (0.68–1.60)	0.847
T3 (≥1.92)	0.85 (0.62–1.16)	0.275	0.91 (0.61–1.35)	0.607
**n−6 PUFA, g/day**				
T1 ( ≤ 12.74)	1.00		1.00	
T2 (12.75–17.08)	1.07 (0.76–1.52)	0.677	1.08 (0.70–1.67)	0.721
T3 (≥17.09)	0.70 (0.53–0.93)	0.016	0.64 (0.47–0.88)	0.009

a *The adjusted variables were age and gender in model 1*.

b *The adjusted variables were age, sex, family income-to-poverty ratio, education level, marital status, ethnicity, platelet count, ALT, AST, GGT, ALP, albumin, total bilirubin, pre-hypertension, hypertension, diabetes, prediabetes, CVD, history of cancer, dyslipidemia, use of oral corticosteroid over 180 days, depression status, smoking status, BMI, waist circumference, regular exercise, HEI-2015, energy intake, sleep duration, and history of sleep disorders in model 2*.

*T1, first tertile; T2, second tertile; T3, third tertile; UFA, unsaturated fatty acid; SFA, saturated fatty acid; MUFA, monounsaturated fatty acid; PUFA, polyunsaturated fatty acid*.

did not show statistically significant association consistently in model 2. Thus, we further explored the association of the main components of dietary fatty acid intakes with significant liver fibrosis risk (Table 3). The ORs (95% CIs) of significant liver fibrosis were 0.69 (0.52–0.91) in model 1 and 0.64 (0.47–0.87) in model 2 for linoleic acid intake (the highest tertile) when compared to the reference tertile. In addition, in only age- and gender-adjusted model 1, for the highest tertile vs. lowest tertile, the ORs (95% CIs) of significant liver fibrosis were 1.44 (1.13–1.84) for arachidonic acid intake and 1.68 (1.19–2.36) for stearic acid intake, which did not show statistically significant association consistently in model 2. Except for the fatty acid components mentioned above, other components did not show the statistically significant association with significant liver fibrosis. Moreover, based on the results of multiple logistic regression, we further observed a stable relationship between dietary fatty acid intakes and significant liver fibrosis by sensitivity analysis. The results of sensitivity analyses (Supplementary Figures S1, S2, Supplementary Tables S1, S2) had the same pattern with that in Tables 2, 3, reinforcing the inverse association of unsaturated to saturated fatty acid ratio, PUFA, and linoleic acid consumptions with significant liver fibrosis risk.

**Table 3:** Odds ratios (ORs) and 95% confidence intervals (CIs) for risk of significant liver fibrosis based on tertiles of dietary intakes of fatty acid components.

	**Significant liver fibrosis**			
	**OR1** **(95% CI)** ^a^	***p-*** **value**	**OR2** **(95% CI)** ^b^	***p*** **-value**
**Linoleic acid (18:2), g/day**				
T1 ( ≤ 12.62)	1.00		1.00	
T2 (12.63–16.92)	1.04 (0.74–1.47)	0.791	1.07 (0.70–1.63)	0.749
T3 (≥16.93)	0.69 (0.52–0.91)	0.011	0.64 (0.47–0.87)	0.008
**Linolenic acid (18:3), g/day**				
T1 ( ≤ 1.24)	1.00		1.00	
T2 (1.25–1.77)	1.03 (0.74–1.43)	0.847	1.06 (0.74–1.52)	0.745
T3 (≥1.78)	0.77 (0.56–1.06)	0.103	0.73 (0.48–1.11)	0.127
**Arachidonic acid (20:4), g/day**				
T1 ( ≤ 0.09)	1.00		1.00	
T2 (0.10–0.17)	0.91 (0.60–1.37)	0.626	0.85 (0.47–1.52)	0.555
T3 (≥0.18)	1.44 (1.13–1.84)	0.007	1.18 (0.80–1.73)	0.373
**Butyric acid (4:0), g/day**				
T1 ( ≤ 0.24)	1.00		1.00	
T2 (0.25–0.46)	0.87 (0.62–1.22)	0.397	0.94 (0.64–1.38)	0.748
T3 (≥0.47)	0.97 (0.60–1.57)	0.900	1.19 (0.64–2.20)	0.561
**Caproic acid (6:0), g/day**				
T1 ( ≤ 0.16)	1.00		1.00	
T2 (0.17–0.30)	0.97 (0.69–1.36)	0.833	1.01 (0.70–1.45)	0.957
T3 (≥0.31)	1.16 (0.69–1.94)	0.549	1.39 (0.69–2.77)	0.329
**Caprylic acid (8:0), g/day**				
T1 ( ≤ 0.14)	1.00		1.00	
T2 (0.15–0.25)	1.04 (0.75–1.43)	0.808	1.16 (0.68–1.97)	0.572
T3 (≥0.26)	1.26 (0.84–1.89)	0.238	1.48 (0.80–2.75)	0.197
**Capric acid (10:0), g/day**				
T1 ( ≤ 0.29)	1.00		1.00	
T2 (0.30–0.51)	1.08 (0.78–1.48)	0.631	1.24 (0.87–1.77)	0.207
T3 (≥0.52)	1.29 (0.82–2.03)	0.258	1.71 (0.87–3.33)	0.110
**Lauric acid (12:00), g/day**				
T1 ( ≤ 0.40)	1.00		1.00	
T2 (0.41–0.79)	1.05 (0.74–1.51)	0.759	1.20 (0.71–2.02)	0.481
T3 (≥0.80)	1.12 (0.75–1.68)	0.551	1.28 (0.78–2.10)	0.299
**Myristic acid (14:00), g/day**				
T1 ( ≤ 1.37)	1.00		1.00	
T2 (1.38-2.17)	1.01 (0.70–1.45)	0.970	1.04 (0.62–1.76)	0.873
T3 (≥2.18)	1.21 (0.75–1.96)	0.404	1.40 (0.78–2.54)	0.242
**Palmitic acid (16:0), g/day**				
T1 ( ≤ 11.33)	1.00		1.00	
T2 (11.34–14.15)	0.87 (0.55–1.36)	0.511	0.82 (0.39–1.74)	0.577
T3 (≥14.16)	1.48 (0.98–2.25)	0.062	1.39 (0.72–2.69)	0.303
**Stearic acid (18:0), g/day**				
T1 ( ≤ 4.68)	1.00		1.00	
T2 (4.69–6.16)	0.85 (0.54–1.33)	0.443	0.69 (0.41–1.17)	0.155
T3 (≥6.17)	1.68 (1.19–2.36)	0.006	1.34 (0.74-−2.43)	0.305
**Palmitoleic acid (16:1), g/day**				
T1 ( ≤ 0.78)	1.00		1.00	
T2 (0.79–1.14)	0.96 (0.66–1.41)	0.840	0.87 (0.52–1.45)	0.558
T3 (≥1.15)	1.43 (0.98–2.08)	0.061	1.02 (0.60–1.73)	0.947
**Oleic acid (18:1), g/day**				
T1 ( ≤ 20.68)	1.00		1.00	
T2 (20.69–25.68)	1.14 (0.68–1.89)	0.595	1.19 (0.65–2.16)	0.554
T3 (≥25.69)	1.25 (0.85–1.85)	0.236	1.06 (0.68–1.67)	0.778

a * The adjusted variables were age and gender in model 1*.

b * The adjusted variables were age, sex, family income-to-poverty ratio, education level, marital status, ethnicity, platelet count, ALT, AST, GGT, ALP, albumin, total bilirubin, pre-hypertension, hypertension, diabetes, prediabetes, CVD, history of cancer, dyslipidemia, use of oral corticosteroid over 180 days, depression status, smoking status, BMI, waist circumference, regular exercise, HEI-2015, energy intake, sleep duration, and history of sleep disorders in model 2*.

*T1, first tertile; T2, second tertile; T3, third tertile*.

### Dose–response analysis

**Figure 2:**
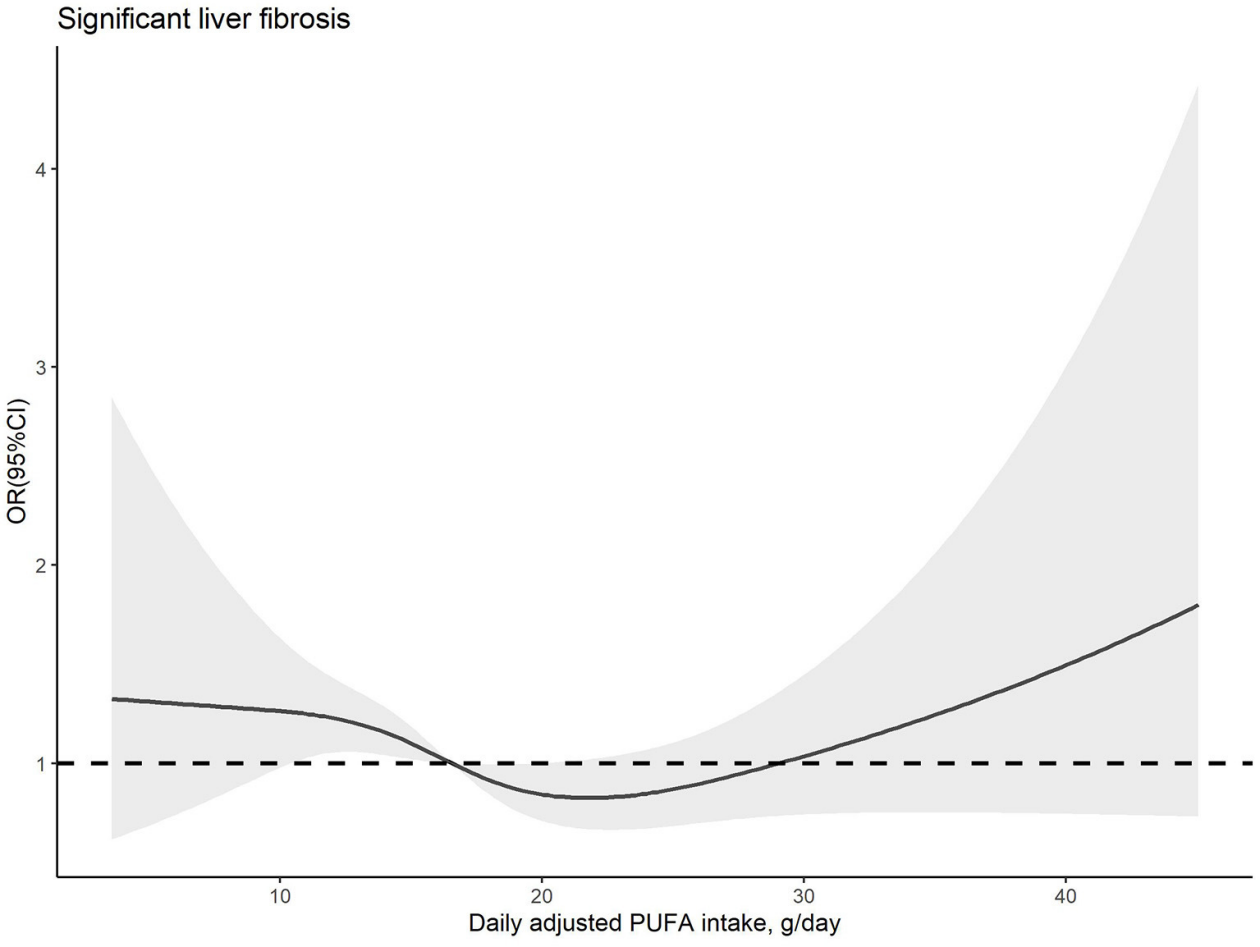
Dose–response relationship between dietary PUFA intake and significant liver fibrosis. The solid line and shadow area represent the estimated odds ratios (ORs) and their corresponding 95% confidence intervals (CIs). The adjusted variables were the same as those in model 2, including age, sex, family income-to-poverty ratio, education level, marital status, ethnicity, platelet count, ALT, AST, GGT, ALP, albumin, total bilirubin, pre-hypertension, hypertension, diabetes, prediabetes, CVD, history of cancer, dyslipidemia, use of oral corticosteroid over 180 days, depression status, smoking status, BMI, waist circumference, regular exercise, HEI-2015, energy intake, sleep duration, and history of sleep disorders.

**Figure 3:**
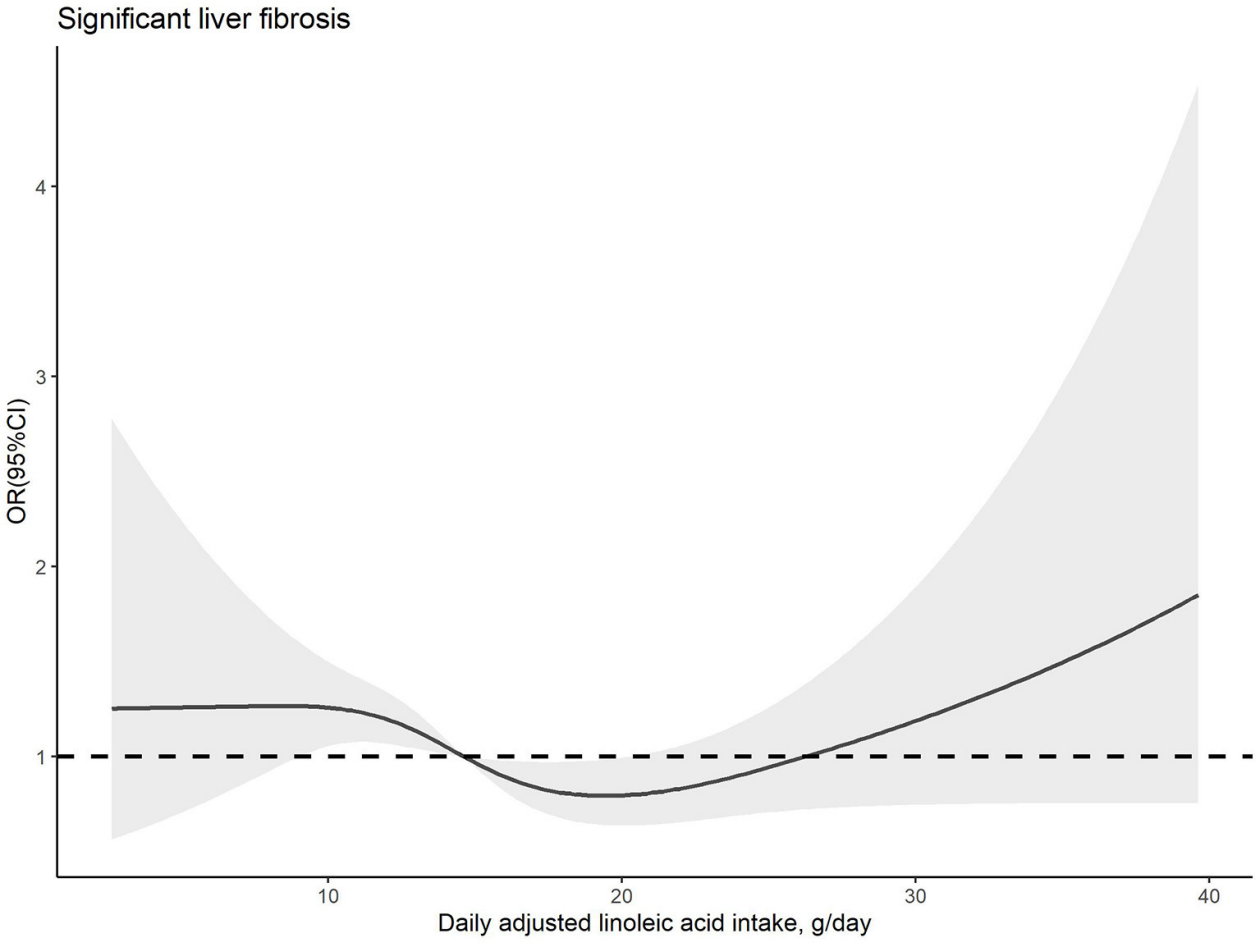
Dose–response relationship between dietary linoleic acid intake and significant liver fibrosis. The solid line and shadow area represent the estimated odds ratios (ORs) and their corresponding 95% confidence intervals (CIs). The adjusted variables were the same as those in model 2, including age, sex, family income-to-poverty ratio, education level, marital status, ethnicity, platelet count, ALT, AST, GGT, ALP, albumin, total bilirubin, pre-hypertension, hypertension, diabetes, prediabetes, CVD, history of cancer, dyslipidemia, use of oral corticosteroid over 180 days, depression status, smoking status, BMI, waist circumference, regular exercise, HEI-2015, energy intake, sleep duration, and history of sleep disorders.

The dose–response relationships between PUFA or linoleic acid intake and the risk of significant liver fibrosis are shown in Figures 2, 3, respectively. The similar U-shaped associations were observed between PUFA or linoleic acid intake and the risk of significant liver fibrosis, showing that the PUFA intake ranging from 16.70 to 19.83 g/day or linoleic acid intake ranging from 14.71 to 20.29 g/day was inversely associated with the risk of significant liver fibrosis, respectively (*p* for non-linearity <0.05).

## Discussion

In this cross-sectional study, after adjusting multiple potential confounders, we pointed out the inverse association between the ratio of UFA to SFA and significant liver fibrosis risk, and further demonstrated the protective factors of total PUFA and its specific component linoleic acid for significant liver fibrosis in general US adults. Sensitivity analysis and RCS analysis showed similar results, reinforcing the significant inverse associations between the UFA to SFA ratio, PUFA, linoleic acid, and significant liver fibrosis risk. However, SFA, UFA, MUFA, and their specific components did not show the statistically significant association with significant liver fibrosis risk.

With the effective treatment available for hepatitis B and C, the worldwide prevalence of NAFLD or non-alcoholic steatohepatitis is currently the leading causes of liver fibrosis (36). Accumulation of excess liver fat is the basis for the contribution of NAFLD; of note, the quality of dietary fatty acids may take a role in the accumulation of liver fat (37, 38). The aforementioned study reported that a hypercaloric SFA-rich diet led to a remarkable increase in hepatic fat; by contrast, a PUFA-enriched diet did not show an increase in hepatic fat (12). Moreover, the 2015–2020 dietary guidelines for Americans recommend replacing total SFA with total UFA and keeping within saturated fat limits, and similar recommendations can also be found in the 2019 Canada's Food Guide (39). Additionally, a recent meta-analysis demonstrated that the replacement of SFA with PUFA reduced the cardiovascular disease risk, and replacement with MUFA was not clear due to the limited data (40). To the best of our knowledge, this study was one of the first studies to explore the association between the UFA to SFA ratio and liver fibrosis risk. As expected, statistically significant inverse association was observed between the UFA to SFA ratio and significant liver fibrosis risk, with a reduction of about 66% in the odds for liver fibrosis risk after adjustment of multiple potential confounders (Table 2). Inconsistent with our findings, an Italian longitudinal study (41), the epidemiologic study referred to the UFA to SFA ratio, indicated that higher UFA to SFA ratio increased total mortality but marginally. Different sample sizes, ethnicity, age, and dietary patterns may be the reasons. Noteworthy, the MUFA intake was 42.1 ± 12.5 g/day, PUFA intake was 7.4 ± 2.6 g/day, and SFA intake was 20.8 ± 7.8 g/day at baseline in the study above, whereas it was 25.34 ± 0.20 g/day, 17.20 ± 0.19 g/day, and 24.12 ± 0.27 g/day, respectively, in this study (Table 1), which implied that the different amount of PUFA or MUFA intakes may influence the healthy effect of UFA to SFA ratio. Furthermore, in this study, PUFA, but not MUFA or SFA, had the statistically significant inverse association with significant liver fibrosis risk, suggesting that PUFA plays a crucial role in the aforementioned relationship.

The n-3 PUFA and n-6 PUFA are the principal series of PUFA, playing the important roles in the development of NAFLD. A cross-sectional study indicated both dietary n-3 and n-6 PUFA had inverse associations with NAFLD risk, using data from NHANES 2007–2014 (42). Additionally, depletion of long-chain PUFA has been reported in non-alcoholic fatty liver (43). Several studies also presented favorable associations between n-3 fatty acid intakes and NAFLD risk (17, 44, 45). Nevertheless, some inconsistent results in terms of n-3 PUFA components were found in more progressive NAFLD, such as fibrosis. In Lytle et al.'s (46) study, it was DHA, but not EPA, that attenuated western diet-linked liver fibrosis by targeting TGF-β pathway, while in another animal study, higher expression of TIMP-1 and TGF-β pro-fibrogenic genes and more severe fibrosis score were found in EPA and DHA together with olive oil-fed mice than that in only olive oil-fed mice (21). Those studies above implied that the different fatty acid components may influence the prevalence of liver fibrosis specifically, due to their different chemical structures and biological effects. However, up to now, limited study has explored the association between fatty acid components and risk of liver fibrosis.

In our study, the components of PUFA, only linoleic acid, presented statistically significant less odds of having significant liver fibrosis. Furthermore, the components of SFA and MUFA were not significantly related to the risk of significant liver fibrosis in model 2, consistent with the result of SFA and MUFA in total. This is inconsistent with a cross-sectional study, which showed the components of SFA lauric acid and myristic acid, palmitoleic and oleic MUFA had inverse associations with liver fibrosis, but a similar association was not observed in higher quartile of the fatty acids above (20). The discrepancies may be partially due to the specific participants with HIV, who has the different nutritional situation from normal people. Moreover, dietary lipid consumptions of total SFA, oleic acid, and linoleic acid had no significant association with the risk of cirrhosis or liver cancer, according to the study using data from NHANES I (47). We suppose that the effects of dietary fatty acids on liver function may vary depending on the intakes of fatty acid components, the stages of liver diseases, and the characteristics of the study population. Thus, we further explored the dose–response association between PUFA or linoleic acid intakes and risk of significant liver fibrosis. The results of RCS predicted that the range estimation of PUFA and linoleic acid intakes for the inversed association with significant liver fibrosis risk.

With the typical Western diet style of higher n-6 PUFA consumption than n-3 PUFA consumption, linoleic acid, as the major n-6 PUFA, can also represent the most consumed fatty acid in PUFA (48). Although linoleic acid is the most consumed PUFA, scarce study has investigated the associations of linoleic acid intake with NAFLD, not to mention liver fibrosis. Only one aforementioned study observed moderate linoleic acid intake increased liver fibrosis risk in subjects with HIV infection (20), and the discrepancy with our study may be due to the special characteristics of participants. Some biological processes may explain the inverse associations between dietary intakes of linoleic acid and significant liver fibrosis partially. Liver fibrosis induced by chronic damage to the liver is related to the accumulation of extracellular matrix (ECM) proteins, which distorts the hepatic wound healing process by forming a fibrous scar, and can proceed to cirrhosis with nodules of regenerating hepatocytes (1). Hepatic stellate cells are the main cell types to produce ECM in liver, which can be triggered by fat-accumulated hepatocytes. Additionally then, activated hepatic stellate cells are migratory and process excessive ECM (49). Furthermore, one randomized controlled trial of 67 participants with abdominally obesity demonstrated that n-6 PUFA reduced liver fat and did not induce inflammation or oxidative stress (50). It is worth to note that the participants in our study were also with higher BMI (29.8 ± 0.3 kg/m^2^) and waist circumference (100.7 ± 0.8 cm), similar to the subjects in the study above. Altogether, we speculate that linoleic acid may reduce the liver fat and thereby reducing the amount of ECM produced by activated hepatic stellate cells, further alleviating the process of liver fibrosis. Because of the observational study limitation, further studies are needed to verify these findings.

This study has several strengths. First, a large-scale and national representative sample was used in our study, which can increase the statistical power and reliability of the findings. Second, we adjusted a large number of potential confounders, including demographic information, laboratory and body measurement parameters, medical conditions, and lifestyle factors. Third, significant liver fibrosis is determined by highly accurate transient elastography, which is considered as the non-invasive standard tool for evaluating significant fibrosis (23). Fourth, we investigated the dose–response relationship between PUFA or linoleic acid intakes and the risk of significant liver fibrosis.

However, the study also includes some limitations. First, our study was a cross-sectional study, which cannot determine the causality between dietary fatty acid intakes and the incidence of significant liver fibrosis. In addition, the dietary data were obtained from two 24-h dietary recalls, and the influence of recall bias was hardly avoided. Finally, we did not perform a stratified analysis due to the limited sample size of participants with significant liver fibrosis, which may hinder its statistical power to clarify associations of dietary fatty acid intakes with significant liver fibrosis risk.

In conclusion, this study observed that the UFA to SFA ratio, dietary PUFA intake, and linoleic acid intake were inversely associated with significant liver fibrosis risk. Furthermore, consumptions of PUFA and specific linoleic acid were in a dose–response relationship with significant liver fibrosis risk, warranting further large-scale prospective studies in this area.

## Data availability statement

The datasets presented in this study can be found in online repositories. The names of the repository/repositories and accession number(s) can be found below: http://www.cdc.gov/nchs/nhanes.htm.

## Ethics statement

The studies involving human participants were reviewed and approved by the National Center for Health Statistics Research Ethics Review Board. The patients/participants provided their written informed consent to participate in this study.

## Author contributions

H-LZ and Z-YL designed the study and performed data analyses and reviewed and editing the manuscript. TZ drafted, reviewed, and edited the manuscript. X-TL contributed in elaborating the tables and figures. All authors contributed to the article and approved the submitted version.

## Funding

This work was supported by the National Natural Science Foundation of China (no. 81973016), Basic and Applied Basic Research Foundation of Guangdong Province (2020A1515110682), and China Postdoctoral Science Foundation (2020M683135).

## Conflict of interest

The authors declare that the research was conducted in the absence of any commercial or financial relationships that could be construed as a potential conflict of interest.

## Publisher's note

All claims expressed in this article are solely those of the authors and do not necessarily represent those of their affiliated organizations, or those of the publisher, the editors and the reviewers. Any product that may be evaluated in this article, or claim that may be made by its manufacturer, is not guaranteed or endorsed by the publisher.

## Abbreviations

CLD: chronic liver diseases
NAFLD: non-alcoholic fatty liver disease
SFA: saturated fatty acids
UFA: unsaturated fatty acids
MUFA: monounsaturated fatty acids
PUFA: polyunsaturated fatty acids
EPA: eicosapentaenoic
DHA: docosahexaenoic
NHANES: National Health and Nutrition Examination Survey
VCTE: vibration controlled transient elastography
LSM: liver stiffness measurement
CAP: controlled attenuation parameter
MEC: Mobile Examination Center
DGAs: Dietary Guidelines for Americans
HEI: healthy eating index
BMI: body mass index
ALT: alanine aminotransferase
ALP: alkaline phosphatase
AST: aspartate aminotransferase
GGT: gamma-glutamyl transferase
HbA1c: glycated hemoglobin A1c
CVD: cardiovascular disease
PHQ-9: Patient Health Questionnaire
SE: standard error
OR: odds ratio
CI: confidence interval
RCS: restricted cubic spline
HIV: human immunodeficiency virus
TGF: transforming growth factor
TIMP: tissue inhibitor of metalloproteinase.
