# Supplementary figures and images for: Dietary linoleic acid and the ratio of unsaturated to saturated fatty acids are inversely associated with significant liver fibrosis risk: A nationwide survey
Source: Front Nutr. 2022 Jul 26;9:938645. doi: 10.3389/fnut.2022.938645 (PMC9360805; doi:10.3389/fnut.2022.938645)

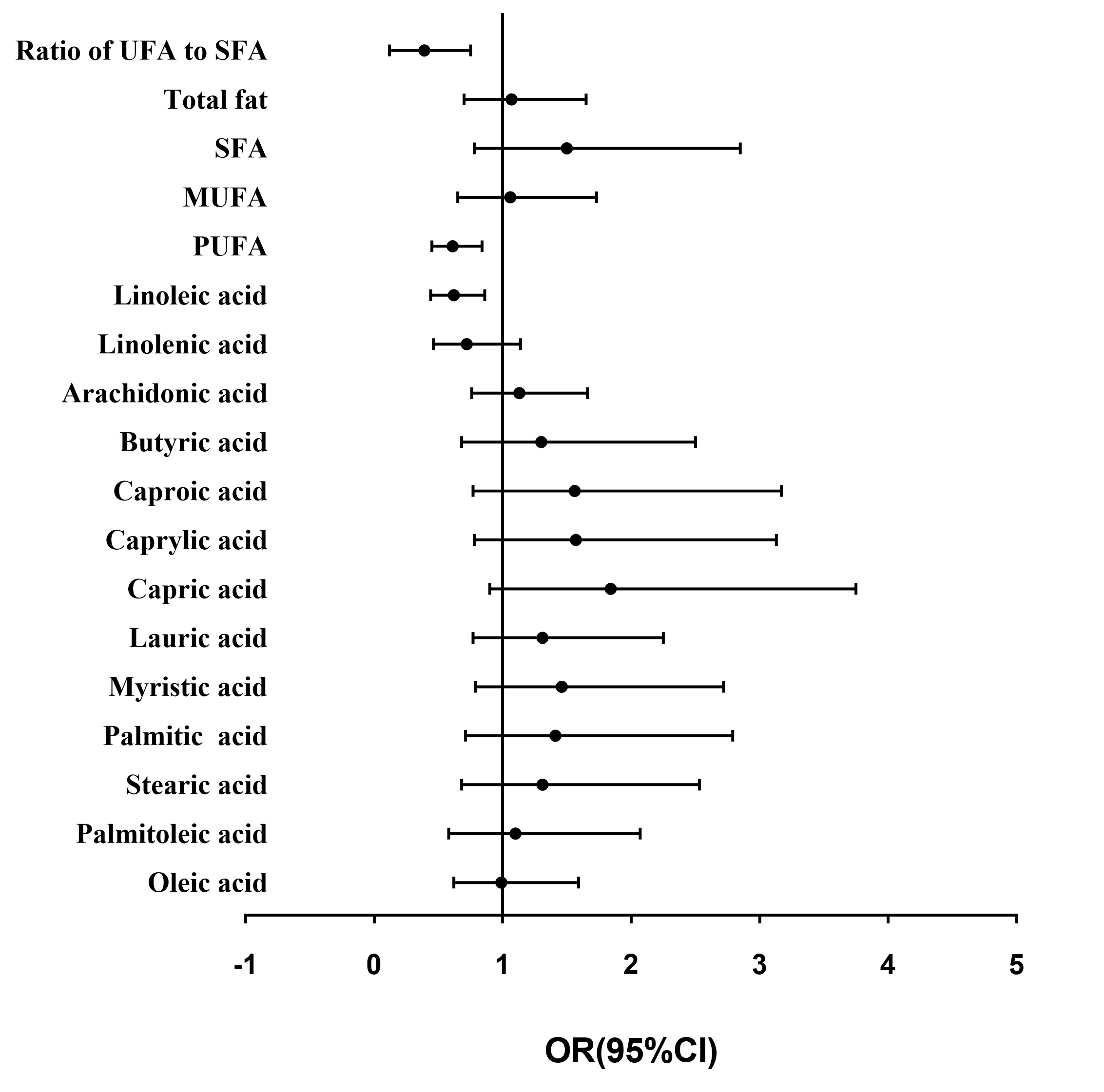

Supplement: Supplementary file 1 [file Image_1.TIF]

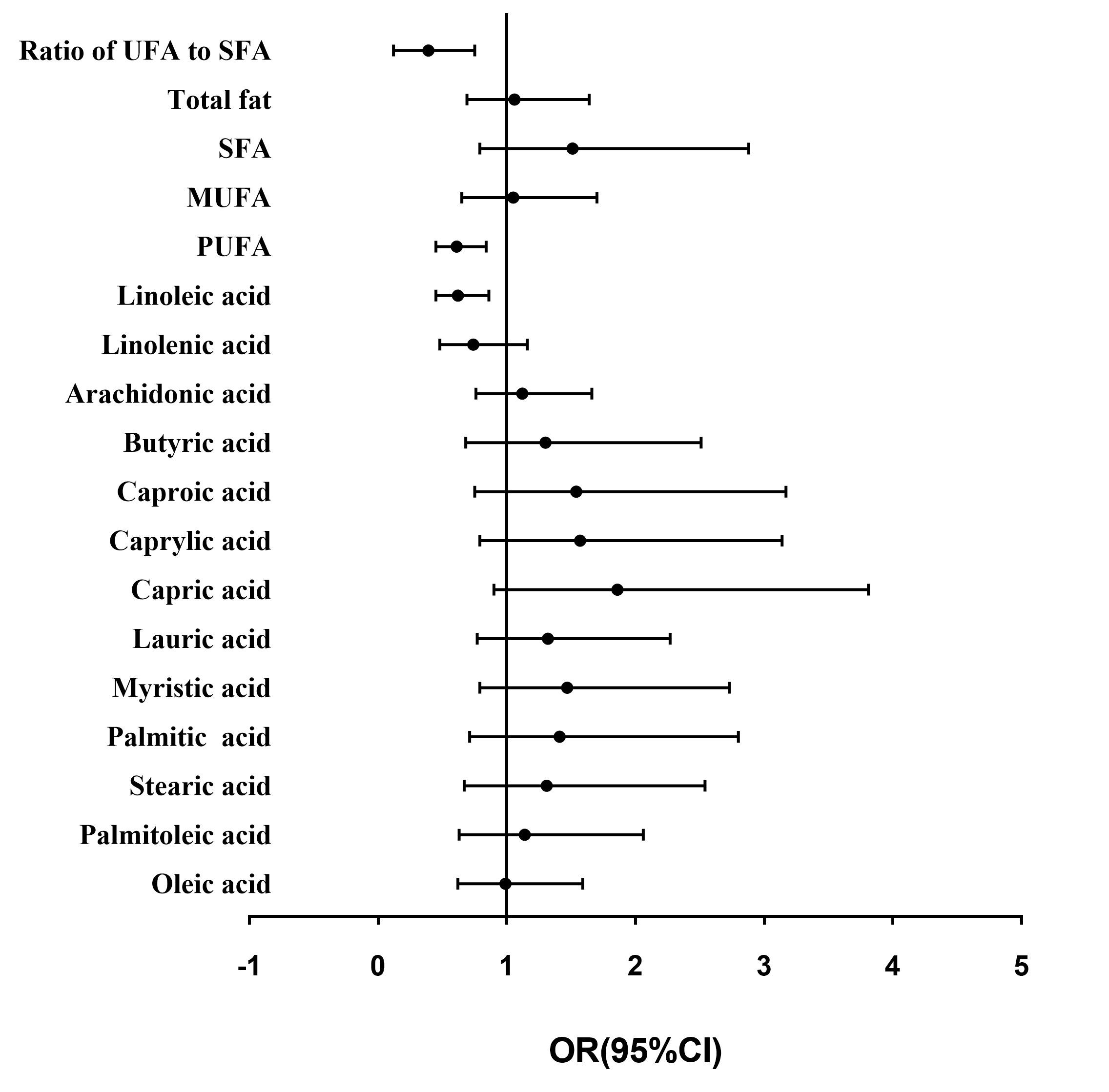

Supplement: Supplementary file 2 [file Image_2.TIF]
