# Supplementary material for: Dietary linoleic acid and the ratio of unsaturated to saturated fatty acids are inversely associated with significant liver fibrosis risk: A nationwide survey
Source: Front Nutr. 2022 Jul 26;9:938645. doi: 10.3389/fnut.2022.938645 (PMC9360805; doi:10.3389/fnut.2022.938645)
Supplement: Supplementary file 3 [file Data_Sheet_1.docx]

Supplementary Material

**Supplementary Table 1.** Multivariate logistic regression model considering dietary fatty acid intakes and the risk of significant liver fibrosis in participants without history of caner, NHANES 2017-2018 (*n* = 3746).

|  | **Significant liver fibrosis** | |
| --- | --- | --- |
|  | **OR1 (95% CI) ^a^** | **OR2 (95% CI) ^b^** |
| **Ratio of UFA to SFA** |  |  |
| ≤1.2 | 1.00 | 1.00 |
| 1.2-2.5 | 0.52 (0.32-0.85) | 0.43 (0.27-0.70) |
| ≥2.5 | 0.48 (0.25-0.92) | 0.30 (0.12-0.75) |
| **Total fat, g/day** |  |  |
| T1 (≤66.29) | 1.00 | 1.00 |
| T2 (66.30-79.08) | 0.99 (0.68-1.45) | 1.14 (0.74-1.75) |
| T3 (≥79.09) | 1.26 (0.89-1.77) | 1.07 (0.70-1.65) |
| **SFA, g/day** |  |  |
| T1 (≤20.03) | 1.00 | 1.00 |
| T2 (20.04-25.62) | 0.96 (0.57-1.62) | 0.97 (0.42-2.26) |
| T3 (≥25.63) | 1.47 (0.95-2.28) | 1.50 (0.78-2.85) |
| **MUFA, g/day** |  |  |
| T1 (≤22.08) | 1.00 | 1.00 |
| T2 (22.09-27.33) | 1.02 (0.63-1.66) | 0.95 (0.54-1.67) |
| T3 (≥27.34) | 1.32 (0.87-2.01) | 1.06 (0.65-1.73) |
| **PUFA, g/day** |  |  |
| T1 (≤14.30) | 1.00 | 1.00 |
| T2 (14.31-19.18) | 1.21 (0.84-1.75) | 1.17 (0.73-1.88) |
| T3 (≥19.19) | 0.72 (0.55-0.94) | 0.61 (0.45-0.84) |
| **Linoleic acid (18:2), g/day** |  |  |
| T1 (≤12.62) | 1.00 | 1.00 |
| T2 (12.63-16.98) | 1.11 (0.75-1.63) | 1.09 (0.68-1.76) |
| T3 (≥16.99) | 0.72 (0.54-0.96) | 0.62 (0.44-0.86) |
| **Linolenic acid (18:3), g/day** |  |  |
| T1 (≤1.24) | 1.00 | 1.00 |
| T2 (1.25-1.76) | 1.05 (0.73-1.49) | 1.01 (0.69-1.48) |
| T3 (≥1.77) | 0.81 (0.60-1.10) | 0.72 (0.46-1.14) |
| **Arachidonic acid (20:4), g/day** |  |  |
| T1 (≤0.10) | 1.00 | 1.00 |
| T2 (0.11-0.17) | 0.87 (0.58-1.31) | 0.79 (0.46-1.38) |
| T3 (≥0.18) | 1.41 (1.06-1.89) | 1.13 (0.76-1.66) |
| **Butyric acid (4:0), g/day** |  |  |
| T1 (≤0.24) | 1.00 | 1.00 |
| T2 (0.25-0.45) | 0.80 (0.54-1.18) | 0.99 (0.65-1.52) |
| T3 (≥0.46) | 0.97 (0.57-1.64) | 1.30 (0.68-2.50) |
| **Caproic acid (6:0), g/day** |  |  |
| T1 (≤0.16) | 1.00 | 1.00 |
| T2 (0.17-0.30) | 0.94 (0.61-1.45) | 1.12 (0.72-1.74) |
| T3 (≥0.31) | 1.16 (0.67-2.02) | 1.56 (0.77-3.17) |
| **Caprylic acid (8:0), g/day** |  |  |
| T1 (≤0.14) | 1.00 | 1.00 |
| T2 (0.15-0.25) | 1.06 (0.71-1.57) | 1.33 (0.72-2.47) |
| T3 (≥0.26) | 1.28 (0.81-2.02) | 1.57 (0.78-3.13) |
| **Capric acid (10:0), g/day** |  |  |
| T1 (≤0.29) | 1.00 | 1.00 |
| T2 (0.30-0.51) | 1.04 (0.70-1.56) | 1.38 (0.91-2.11) |
| T3 (≥0.52) | 1.26 (0.75-2.13) | 1.84 (0.90-3.75) |
| **Lauric acid (12:00), g/day** |  |  |
| T1 (≤0.40) | 1.00 | 1.00 |
| T2 (0.41-0.79) | 1.05 (0.70-1.59) | 1.25 (0.69-2.24) |
| T3 (≥0.80) | 1.13 (0.73-1.76) | 1.31 (0.77-2.25) |
| **Myristic acid (14:00), g/day** |  |  |
| T1 (≤1.37) | 1.00 | 1.00 |
| T2 (1.38-2.17) | 0.95 (0.62-1.47) | 1.06 (0.57-1.98) |
| T3 (≥2.18) | 1.19 (0.72-1.96) | 1.46 (0.79-2.72) |
| **Palmitic acid (16:0), g/day** |  |  |
| T1 (≤11.34) | 1.00 | 1.00 |
| T2 (11.35-14.20) | 0.95 (0.58-1.56) | 0.87 (0.39-1.91) |
| T3 (≥14.21) | 1.49 (0.94-2.37) | 1.41 (0.71-2.79) |
| **Stearic acid (18:0), g/day** |  |  |
| T1 (≤4.67) | 1.00 | 1.00 |
| T2 (4.68-6.17) | 0.81 (0.51-1.29) | 0.65 (0.35-1.20) |
| T3 (≥6.18) | 1.61 (1.09-2.38) | 1.31 (0.68-2.53) |
| **Palmitoleic acid (16:1), g/day** |  |  |
| T1 (≤0.78) | 1.00 | 1.00 |
| T2 (0.79-1.15) | 1.16 (0.77-1.74) | 1.13 (0.66-1.92) |
| T3 (≥1.16) | 1.50 (0.96-2.35) | 1.10 (0.58-2.07) |
| **Oleic acid (18:1), g/day** |  |  |
| T1 (≤20.72) | 1.00 | 1.00 |
| T2 (20.73-25.70) | 1.06 (0.63-1.78) | 1.02 (0.56-1.87) |
| T3 (≥25.71) | 1.23 (0.81-1.85) | 0.99 (0.62-1.59) |

^a^: The adjusted variables were age and gender in model 1.

^b^: The adjusted variables were age, sex, family income-to-poverty ratio, education level, marital status, ethnicity, platelet count, ALT, AST, GGT, ALP, albumin, total bilirubin, pre-hypertension, hypertension, diabetes, prediabetes, CVD, history of cancer, dyslipidemia, use of oral corticosteroid over 180 days, depression status, smoking status, BMI, waist circumference, regular exercise, HEI-2015, energy intake, sleep duration, history of sleep disorders in model 2.

Abbreviations: T1, first tertile; T2, second tertile; T3, third tertile; UFA, unsaturated fatty acid; SFA, saturated fatty acid; MUFA, monounsaturated fatty acid; PUFA, polyunsaturated fatty acid.

**Supplementary Table 2.** Multivariate logistic regression model considering dietary fatty acid intakes and the risk of significant liver fibrosis in participants without history of caner and without oral corticosteroid intake over 180 days, NHANES 2017-2018 (*n* = 3715).

|  | **Significant liver fibrosis** | |
| --- | --- | --- |
|  | **OR1 (95% CI) ^a^** | **OR2 (95% CI) ^b^** |
| **Ratio of UFA to SFA** |  |  |
| ≤1.2 | 1.00 | 1.00 |
| 1.2-2.5 | 0.52 (0.32-0.85) | 0.43 (0.27-0.70) |
| ≥2.5 | 0.48 (0.25-0.92) | 0.30 (0.12-0.75) |
| **Total fat, g/day** |  |  |
| T1 (≤66.45) | 1.00 | 1.00 |
| T2 (66.46-79.24) | 1.00 (0.69-1.44) | 1.13 (0.73-1.75) |
| T3 (≥79.25) | 1.27 (0.90-1.78) | 1.06 (0.69-1.64) |
| **SFA, g/day** |  |  |
| T1 (≤20.08) | 1.00 | 1.00 |
| T2 (20.09-25.67) | 0.97 (0.58-1.63) | 0.97 (0.42-2.26) |
| T3 (≥25.68) | 1.49 (0.96-2.31) | 1.51 (0.79-2.88) |
| **MUFA, g/day** |  |  |
| T1 (≤ 22.15) | 1.00 | 1.00 |
| T2 (22.16-27.40) | 1.02 (0.63-1.65) | 0.95 (0.53-1.69) |
| T3 (≥27.41) | 1.33 (0.87-2.02) | 1.05 (0.65-1.70) |
| **PUFA, g/day** |  |  |
| T1 (≤14.32) | 1.00 | 1.00 |
| T2 (14.33-19.22) | 1.21 (0.84-1.74) | 1.17 (0.73-1.86) |
| T3 (≥19.23) | 0.72 (0.55-0.94) | 0.61 (0.45-0.84) |
| **Linoleic acid (18:2), g/day** |  |  |
| T1 (≤12.64) | 1.00 | 1.00 |
| T2 (12.65-17.01) | 1.11 (0.76-1.61) | 1.09 (0.68-1.75) |
| T3 (≥17.02) | 0.71 (0.54-0.95) | 0.62 (0.45-0.86) |
| **Linolenic acid (18:3), g/day** |  |  |
| T1 (≤1.24) | 1.00 | 1.00 |
| T2 (1.25-1.77) | 1.01 (0.71-1.44) | 0.98 (0.66-1.45) |
| T3 (≥1.78) | 0.83 (0.62-1.12) | 0.74 (0.48-1.16) |
| **Arachidonic acid (20:4), g/day** |  |  |
| T1 (≤0.10) | 1.00 | 1.00 |
| T2 (0.11-0.17) | 0.87 (0.58-1.30) | 0.79 (0.46-1.38) |
| T3 (≥0.18) | 1.42 (1.07-1.89) | 1.12 (0.76-1.66) |
| **Butyric acid (4:0), g/day** |  |  |
| T1 (≤0.24) | 1.00 | 1.00 |
| T2 (0.25-0.45) | 0.81 (0.55-1.19) | 1.00 (0.66-1.51) |
| T3 (≥0.46) | 0.98 (0.58-1.66) | 1.30 (0.68-2.51) |
| **Caproic acid (6:0), g/day** |  |  |
| T1 (≤0.16) | 1.00 | 1.00 |
| T2 (0.17-0.30) | 0.97 (0.63-1.49) | 1.15 (0.74-1.78) |
| T3 (≥0.31) | 1.15 (0.65-2.01) | 1.54 (0.75-3.17) |
| **Caprylic acid (8:0), g/day** |  |  |
| T1 (≤0.14) | 1.00 | 1.00 |
| T2 (0.15-0.25) | 1.07 (0.72-1.58) | 1.34 (0.72-2.49) |
| T3 (≥0.26) | 1.28 (0.82-2.00) | 1.57 (0.79-3.14) |
| **Capric acid (10:0), g/day** |  |  |
| T1 (≤0.29) | 1.00 | 1.00 |
| T2 (0.30-0.51) | 1.06 (0.71-1.57) | 1.39 (0.92-2.11) |
| T3 (≥0.52) | 1.27 (0.75-2.15) | 1.86 (0.90-3.81) |
| **Lauric acid (12:00), g/day** |  |  |
| T1 (≤0.40) | 1.00 | 1.00 |
| T2 (0.41-0.79) | 1.06 (0.70-1.61) | 1.25 (0.69-2.26) |
| T3 (≥0.80) | 1.13 (0.73-1.76) | 1.32 (0.77-2.27) |
| **Myristic acid (14:00), g/day** |  |  |
| T1 (≤1.37) | 1.00 | 1.00 |
| T2 (1.38-2.18) | 0.96 (0.63-1.48) | 1.08 (0.58-2.00) |
| T3 (≥2.19) | 1.21 (0.73-2.01) | 1.47 (0.79-2.73) |
| **Palmitic acid (16:0), g/day** |  |  |
| T1 (≤11.38) | 1.00 | 1.00 |
| T2 (11.39-14.24) | 0.95 (0.58-1.56) | 0.87 (0.39-1.92) |
| T3 (≥14.25) | 1.49 (0.94-2.38) | 1.41 (0.71-2.80) |
| **Stearic acid (18:0), g/day** |  |  |
| T1 (≤4.69) | 1.00 | 1.00 |
| T2 (4.70-6.19) | 0.81 (0.51-1.29) | 0.65 (0.35-1.20) |
| T3 (≥6.20) | 1.62 (1.09-2.39) | 1.31 (0.67-2.54) |
| **Palmitoleic acid (16:1), g/day** |  |  |
| T1 (≤0.78) | 1.00 | 1.00 |
| T2 (0.79-1.15) | 1.21 (0.81-1.81) | 1.18 (0.71-1.97) |
| T3 (≥1.16) | 1.57 (1.01-2.43) | 1.14 (0.63-2.06) |
| **Oleic acid (18:1), g/day** |  |  |
| T1 (≤20.76) | 1.00 | 1.00 |
| T2 (20.77-25.78) | 1.06 (0.65-1.74) | 1.02 (0.56-1.86) |
| T3 (≥25.79) | 1.23 (0.82-1.84) | 0.99 (0.62-1.59) |

^a^: The adjusted variables were age and gender in model 1.

^b^: The adjusted variables were age, sex, family income-to-poverty ratio, education level, marital status, ethnicity, platelet count, ALT, AST, GGT, ALP, albumin, total bilirubin, pre-hypertension, hypertension, diabetes, prediabetes, CVD, history of cancer, dyslipidemia, use of oral corticosteroid over 180 days, depression status, smoking status, BMI, waist circumference, regular exercise, HEI-2015, energy intake, sleep duration, history of sleep disorders in model 2.

Abbreviations: T1, first tertile; T2, second tertile; T3, third tertile; UFA, unsaturated fatty acid; SFA, saturated fatty acid; MUFA, monounsaturated fatty acid; PUFA, polyunsaturated fatty acid.

# Supplementary Figure captions

**Supplementary Figure 1.** Sensitivity analysis of the association between dietary fatty acid intakes and the risk of significant liver fibrosis excluding participants with history of cancer.

The adjusted variables were the same as those in model 2, including age, sex, family income-to-poverty ratio, education level, marital status, ethnicity, platelet count, ALT, AST, GGT, ALP, albumin, total bilirubin, pre-hypertension, hypertension, diabetes, prediabetes, CVD, history of cancer, dyslipidemia, use of oral corticosteroid over 180 days, depression status, smoking status, BMI, waist circumference, regular exercise, HEI-2015, energy intake, sleep duration, history of sleep disorders.

Abbreviations: UFA, unsaturated fatty acid; SFA, saturated fatty acid; MUFA, monounsaturated fatty acid; PUFA, polyunsaturated fatty acid.

**Supplementary Figure 2.** Sensitivity analysis of the association between dietary fatty acid intakes and the risk of significant liver fibrosis excluding participants with history of cancer and with use of oral corticosteroid over 180 days.

The adjusted variables were the same as those in model 2, including age, sex, family income-to-poverty ratio, education level, marital status, ethnicity, platelet count, ALT, AST, GGT, ALP, albumin, total bilirubin, pre-hypertension, hypertension, diabetes, prediabetes, CVD, history of cancer, dyslipidemia, use of oral corticosteroid over 180 days, depression status, smoking status, BMI, waist circumference, regular exercise, HEI-2015, energy intake, sleep duration, history of sleep disorders.

Abbreviations: UFA, unsaturated fatty acid; SFA, saturated fatty acid; MUFA, monounsaturated fatty acid; PUFA, polyunsaturated fatty acid.
